# A Lesson to Parents

**Published:** 1877-11

**Authors:** 


### A LESSON TO PARENTS.

I HAD been married fifteen years. Three beautiful daughters enlivened the domestic hearth, the youngest of whom was in her eighth year. A more happy and contented household was no where to be found. My wife was amiable, intelligent, and contented. We were not wealthy; but Providence had preserved us from want; and we had learned that "contentment without wealth, is better than wealth without contentment."

It was my custom, when returning home at night, to drop into one of the many shops that are constantly open in the business streets of the metropolis, and purchase some trifling dainties, such as fruit or confectionery, to present to mother and the children. I need not say how delighted the little ones were at this slight expression of paternal consideration. On one occasion I had purchased some remarkably fine apples. After the repast, half a dozen were left untouched, and my thrifty companion forthwith removed them to the place of deposit, where it was her custom to preserve the remains of our nick-nacks. A day or two after, when I had seated myself at the table to dine, she said to me smilingly:

"So, father has found the way to my safety-box, has he?"

I was at a loss to understand the meaning, and desired her to explain.

"Have you not been in my drawer?"

"What drawer?"

"The upper drawer in my chamber bureau. Did you not take therefrom the largest of the pippins I had put away for the girls?"

"No—I did not!"

"You did not?"

"Not I! I have not seen an apple since the evening I purchased them."

A slight cloud passed over the countenance of my wife. She was troubled. The loss of the apple was in itself nothing; but we had carefully instructed our children not to appropriate to their use, any article whatever of family consumption, without permission; and as permission, when the demand was at all reasonable, had never been denied them, she was loth to suspect any one of them of the offense. We had a servant-girl in the family, but as she was supposed to know nothing of the apples, my wife hesitated to charge it upon her. She at length broke the silence by saying:

"We must examine the affair. I can hardly think one of the children would so act. If we find them guilty, we must reprove them. Will you please look into it?"

The girls were separately called into my presence; the eldest first.

"Eliza, did you take from your mother's drawer, an apple?"

"No, sir."

"Maria, did you take from your mother's drawer, an apple?"

"No, sir."

"Mary, did you take from your mother's drawer, an apple?"

"No, sir."

"It must have been taken by the servant; call her to me," said I, addressing my wife.

"Nell, how came you to take from the drawer of your mistress, without permission, the largest of the apples she had placed there?"

"Wot apples?"

"Did you take no apple from the drawer of your mistress?"

"No sa."

Now, it was evident that falsehood existed somewhere. Could it be that one of my children had told me a lie? The thought harassed me. I was not able to attend to business. I went to the store—but soon returned again. Meanwhile, the servant-girl had communicated to her mistress that she had seen our youngest go into the garret with a large apple, the morning before. On examination, the core, and several pieces of the rind were found upon the floor. I again called Mary to me, and said to her affectionately:

"Mary, my daughter, did you not go into the garret yesterday?"

"Yes, sir."

"Did you go there with an apple?"

"No, sir."

"Did you notice any thing on the floor?"

"No, sir."

I was unwilling to believe my sweet child capable of telling me a falsehood; but appearances were against her. The fault lay between her and the servant, and while I was desirous to acquit my child, I did not wish to accuse unjustly the negro. I therefore took Mary into a room alone, I spoke to her of the enormity of lying—of the necessity of telling the truth—of the severe punishment I should be compelled to inflict upon her, if she did not confess the whole to me, and with tears in my eyes urged her to say that she had done it, if indeed she had. Gradually, I became convinced of her guilt; and now I felt determined she *should* confess it. My threatenings were not without effect. After weeping and protesting her innocence, and weeping and again protesting, my threatenings seemed to alarm her, and falling upon her knees, she said: "Father, I *did* take the apple."

Never shall I forget that moment. My child confessed that she was a liar, in my presence!

Suppressing my emotion, I retired; and Mary, rising from her position, ran to her mother, and in a paroxysm of grief cried out:

"Mother, I did *not* take the apple. But father has made me confess that I did."

Here was a new aspect of affairs. Lie multiplied upon lie. Could it be possible! My dear Mary, who had never been known to deceive us—so affectionate—so gentle—so truthful in all the past—could it be possible that she was a confirmed liar! Necessity was stronger than the tenderness of the father. I chastised her for the first time in my life—severely, severely chastised her! It almost broke her heart—and I may add, it almost broke mine also.

Yet Mary was innocent! After-events proved that the negro was the thief. She had conjured up the story of the garret, knowing that Mary would not deny having been there, and to make the circumstances strong against her, had strewn apple-rinds on the floor. I never think of the event without tears. But it has taught me a useful lesson, and that is never to threaten a child into a lie, when it may be he is telling the truth. The only lie I ever knew Mary to tell me, I myself forced upon her by threatenings. It has also fixed in my mind the determination to employ no servant in my family, when I can possibly do without.

